# Modified Histone Peptides Linked to Magnetic Beads Reduce Binding Specificity

**DOI:** 10.3390/ijms23031691

**Published:** 2022-02-01

**Authors:** Jenna N. Meanor, Albert J. Keung, Balaji M. Rao

**Affiliations:** 1 Department of Chemical and Biomolecular Engineering, North Carolina State University, Campus Box 7905, Raleigh, NC 27606, USA; jnmeanor@ncsu.edu; 2 Golden LEAF Biomanufacturing Training and Education Center (BTEC), North Carolina State University, Raleigh, NC 27695, USA

**Keywords:** yeast surface display, histone post-translational modifications, affinity reagents, protein engineering, chromodomain, binder, reader, antibody, epigenome engineering, synthetic biology

## Abstract

Histone post-translational modifications are small chemical changes to the histone protein structure that have cascading effects on diverse cellular functions. Detecting histone modifications and characterizing their binding partners are critical steps in understanding chromatin biochemistry and have been accessed using common reagents such as antibodies, recombinant assays, and FRET-based systems. High-throughput platforms could accelerate work in this field, and also could be used to engineer de novo histone affinity reagents; yet, published studies on their use with histones have been noticeably sparse. Here, we describe specific experimental conditions that affect binding specificities of post-translationally modified histones in classic protein engineering platforms and likely explain the relative difficulty with histone targets in these platforms. We also show that manipulating avidity of binding interactions may improve specificity of binding.

## 1. Introduction

Post-translational modifications (PTMs) of histone proteins play pivotal roles in orchestrating the chromatin function, including in DNA repair, gene transcription, and cell replication [1,2,3,4]. The characterization of natural binders of histone PTMs as well as the generation of engineered affinity reagents such as antibodies have advanced our understanding of chromatin biology [1,5,6,7,8,9,10,11,12,13,14]. However, despite the importance of molecular interactions with histone PTMs, the current processes of characterizing them and engineering new affinity reagents are typically laborious, low-throughput, and often result in reagents with variable specificity [15]. This is despite the fact that high-throughput platforms exist for characterizing and engineering proteins more generally, including platforms such as yeast surface display, phage display, and mRNA display, as well as high-throughput screening techniques such as magnetic activated cell sorting (MACS) and fluorescence-activated cell sorting [16,17]. 

This gap in the literature surrounding the engineering of affinity reagents for histone PTMs should perhaps not be unexpected. Modified histone tails are associated with high charge density and low overall hydrophobicity [18,19,20]. Another limiting factor is the high density of basic residues in histone proteins which has previously been shown to increase the level of nonspecific binding in protein–protein interactions more generally [9]. For example, proteins such as the SARS-CoV N protein and evolved antibodies have exhibited nonspecific protein–protein interactions due to an enrichment in basic residues [21,22,23]. Finally, the N-terminal tails of histone proteins exhibit intrinsic “natively unfolded” states making them difficult to use in protein engineering platforms [18]. This collection of challenging molecular properties may negatively impact how histone peptides interact not only with target molecules, but also with organic and inorganic components of protein engineering platforms. 

Here, we identify a critical limitation in the conventional workflow of some of these platform approaches that may explain their underutilization in the context of histone PTMs [24]. The isolation or identification of histone-binding proteins using platforms such as yeast surface display typically rely on biotin-mediated immobilization of histone PTM targets on magnetic beads for subsequent panning and magnetic separation of putative binders. Through a series of experiments, we show that immobilization of biotinylated peptides on streptavidin-functionalized magnetic beads results in the loss of specificity of binding to histone PTMs. We then present an alternative strategy that may alleviate the problems arising from peptide immobilization. 

## 2. Results

### 2.1. Yeast Surface Display Provides a Facile Platform to Characterize Histone Reader Specificities

**Figure 1:**
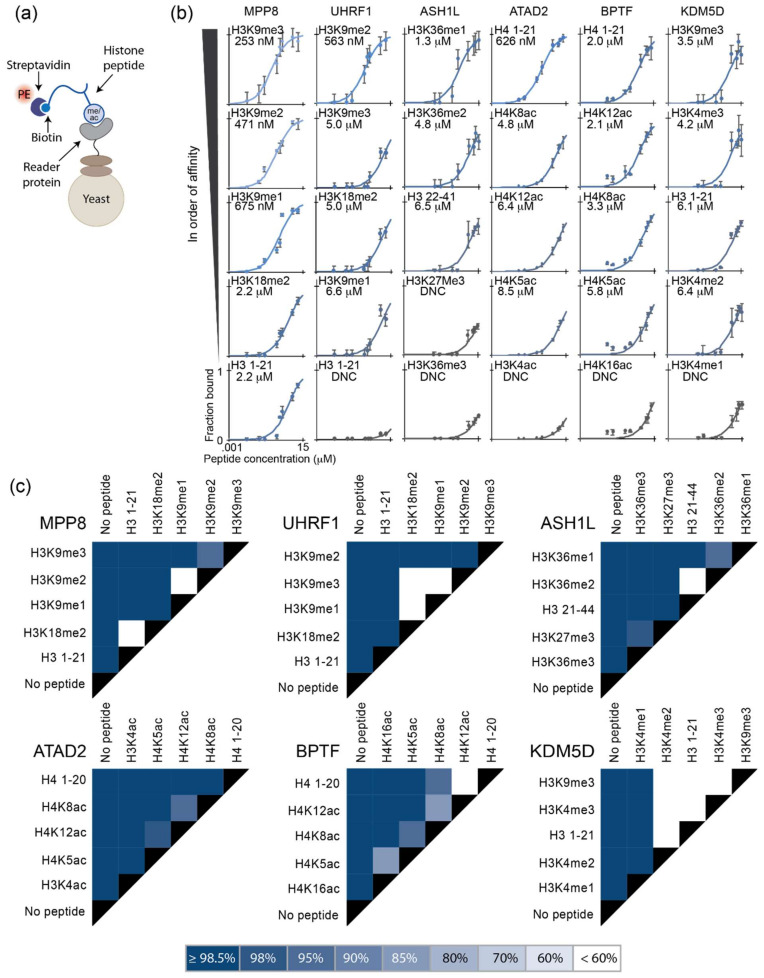
Yeast surface display provides a facile platform to characterize histone reader specificity. (**a**) Interaction between the biotinylated peptide and the displayed reader protein is measured via streptavidin–PE. (**b**) Binding isotherms and calculated binding affinities of the displayed reader proteins to the respective peptides; error bars represent the standard deviation from triplicate samples. (**c**) Binding affinity discrimination determined by the overlap of confidence intervals. More distinct binding affinities (K_D_s) exhibit higher-percentage confidence intervals that do not overlap. Darker colors are associated with a higher level of discrimination between the respective peptides. The legend indicates the confidence intervals at which binding affinities were distinguishable.

For this study, we chose six protein domains with a diverse range of specificities and affinities for histone PTMs as reported in the literature: the chromodomain of MPP8, the tandem Tudor domains of UHRF1, the bromo-adjacent homology domain of ASH1L, the bromodomains of ATAD2 and BPTF, and the jmjN domain of KDM5D (Table 1, Supplemental Table S1). We first asked if the binding specificities and relative affinities of these natural binding domains could be readily characterized in a semi-high-throughput fashion, without the need for recombinant protein production and purification. We leveraged the yeast surface display technology to present the protein domains and mixed yeast with soluble synthetic peptides with PTMs to quantitatively assess binding specificity (Supplemental Table S2). Briefly, yeast cells displaying one of the six proteins were incubated with a titration series of modified histone peptides that were also biotinylated to provide a handle for fluorescent labeling. At each peptide concentration, the fraction of the displayed protein bound to the soluble peptide was determined by streptavidin–PE labeling of the biotinylated peptide through flow cytometry (Figure 1a, Supplemental Table S3). The data were fit to a monovalent binding isotherm to estimate apparent equilibrium dissociation constants as previously described [16] (Figure 1b,c). For all the proteins, the binding data followed the expected binding trends based on the previous literature (Table 1), suggesting this as a facile method for characterizing the binding of proteins to histone peptides with PTMs. 

### 2.2. Discrimination of Binding Specificity and Detection of Weak Binders Are Abrogated in MACS

The individual labeling of yeast displaying histone-binding proteins results in specific and clean results and provides a facile and accessible surrogate approach to characterize binding specificity and relative affinity of proteins to histone PTMs. However, scaling this type of characterization to many proteins or panning for specific histone binders from a diverse library of protein candidates requires a different approach; typically, peptides are immobilized on magnetic beads and used to pan binders from a library of candidates (i.e., magnetic activated cell sorting or MACS). Therefore, we next asked if we could identify a potential reason this high-throughput approach has not been widely implemented, or at least reported, previously. 

**Figure 2:**
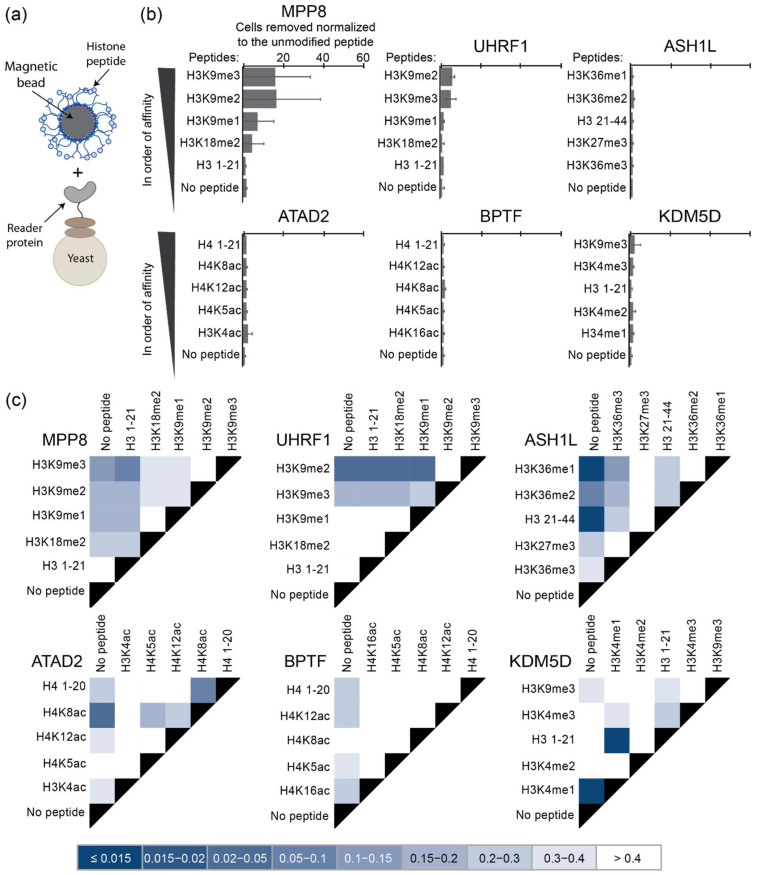
Discrimination of binding specificity and detection of weak binders are abrogated in MACS. (**a**) Interaction between the peptide-coated magnetic beads and the yeast-displaying reader protein. (**b**) Relative amount of yeast-displaying reader proteins magnetically separated by beads linked to modified histone peptides compared to an unmodified histone peptide control; error bars represent the standard deviation from triplicate samples; peptides displayed in order of binding affinity calculated in Figure 1 and from the literature (see Table 1). (**c**) Discrimination between the amounts of yeast separated via magnetization; darker colors are associated with a higher level of discrimination; the legend indicates the p-value comparing each peptide; pairwise comparison via single-factor ANOVA.

To do this, we first tested if the selected protein domains retained PTM-specific binding when the biotinylated histone peptides were immobilized on streptavidin-coated magnetic beads rather than freely presented in the solution (Figure 2a). These beads were mixed with yeasts that both displayed the binder proteins and an engineered luciferase reporter, NanoLuc. The beads and the bound yeast were then pulled down using a magnet. NanoLuc allowed for quantification via luminescence as previously described by Bacon et al. [35]. Based on a recent quantitative yeast–yeast two-hybrid system, the relative amount of yeast pulled down from the system by each modified histone peptide should rely solely on the strength of the interaction [35]; the lower the K_D_, the more yeast should be removed. The yeasts displaying just luciferase were also tested as the negative control and exhibited a similar background to the yeasts displaying proteins mixed with nontarget histone peptides (Supplemental Figure S1). 

Interestingly, the number of cells pulled down by each histone peptide, normalized to the number pulled down with an unmodified control histone peptide, did not match the expected trends in relative affinity (Figure 2b, Table 1). For those proteins with stronger overall affinity to modified histone peptides, such as MPP8 and UHRF1, MACS was unable to distinguish between closely related PTMs. Specifically, for both MPP8 and UHRF1, classic MACS was unable to discriminate binding between H3K9me2 and H3K9me3 (Figure 2c). Furthermore, there was no discernable pattern for proteins with weaker affinity to their respective PTMs (ASH1L, ATAD2, BRTF, KDM5D), potentially suggesting limitations of this platform to both discriminate binding to specific histone peptides as well as capture low-affinity binders in general. The stringency and specificity of pulldown assays are commonly controlled by tuning buffer conditions. We therefore screened a wide range of buffer conditions that varied surfactant and protein concentrations, ionic strength, and yeast-to-bead ratios. Despite testing many distinct conditions informed by the literature [16,31,36,37,38], we observed no significant improvement in the binding specificity and ability to capture weak-affinity binders (Supplemental Figure S2).

### 2.3. Antibodies Label More Specifically When Histone Peptides Are Presented on Yeast versus Bead Surfaces 

**Figure 3:**
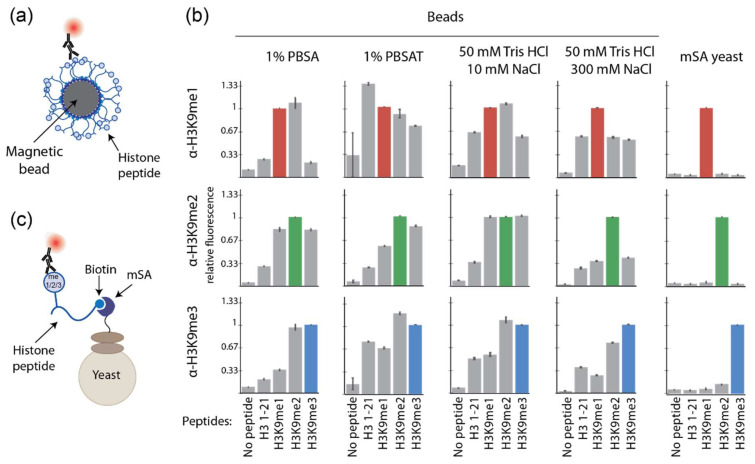
Antibodies label more specifically when histone peptides are presented on yeast versus bead surfaces. (**a**) Interaction between the peptide-coated magnetic beads and the corresponding primary and secondary antibodies. (**b**) Relative fluorescence of each peptide–antibody pairing. The pairings with expected specific interactions with each antibody are indicated by colored bars; error bars represent the standard deviation from triplicate samples. (**c**) Interaction between yeast displaying mSA, biotinylated peptide, and the corresponding primary and secondary antibodies.

We hypothesized that linking peptides to the surface of the beads might be negatively affecting binding specificity. We therefore further challenged peptide-linked beads with a distinct and widely used set of affinity reagents, antibodies, and found they also exhibited poor binding specificity. Streptavidin-coated magnetic beads were first linked to biotinylated peptides containing unmodified, mono-, di-, or trimethylated lysine 9, and then with the corresponding primary and secondary antibodies to each specific modification (Figure 3a, Supplemental Table S4), followed by detection by flow cytometry. In all the conditions tested, antibodies cross-reacted significantly and nonspecifically with all four histone peptides (Figure 3b, Supplemental Figure S3). 

While these antibodies were chosen for their widespread use in many publications [39,40,41,42,43], we considered that the antibodies themselves may lack specificity; however, we found that the antibodies were indeed specific when histone peptides were displayed in a different context. Specifically, when the same set of peptides was linked to the surface of yeast (instead of magnetic beads) through the display of modified monovalent streptavidin (mSA) [44,45,46] (Figure 3c), the same antibodies were able to specifically bind to their target epitope and showed significantly less binding to nontarget epitopes (Figure 3b, right). While the diameters of *Saccharomyces cerevisiae* and the streptavidin-coated magnetic beads used in these experiments were of the same order of magnitude, 5 µm and 2.8 µm, respectively, the amounts of the displayed peptides were not. *S. cerevisiae* can display between 30,000 and 50,000 proteins of interest using the Aga1p and Aga2p display system while magnetic beads can present up to 2 million peptides [16]. This could potentially result in a large avidity effect, masking the ability to distinguish between small differences between histone peptide modifications on beads [47,48].

### 2.4. Decreasing Peptide Density on Beads Does Not Rescue Antibody Labeling Specificity

**Figure 4:**
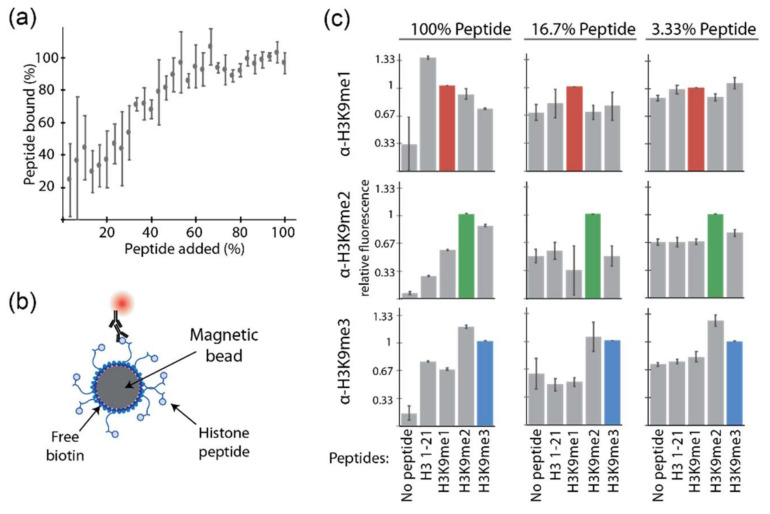
Decreasing peptide density on beads does not rescue antibody labeling specificity. (**a**) Range of the H3K9me2 peptide coverage on magnetic beads was achieved by changing the free biotin to biotinylated peptide ratio. Peptide bound percentage was measured via flow cytometry; error bars represent the standard deviation from triplicate samples. (**b**) Interaction between low-peptide-density magnetic beads and the corresponding primary and secondary antibodies. (**c**) Relative fluorescence of each peptide–antibody pairing. The pairings with expected specific interactions with each antibody are indicated by colored bars. The buffer used was 1% PBSAT; error bars represent the standard deviation from triplicate samples.

To try and mimic the lower density of peptides achievable on yeast, free biotin and biotinylated H3K9me2 peptide were added in increasing ratios to streptavidin-coated magnetic beads while keeping the total biotin content the same; the peptide density on the surface of magnetic beads could be reliably decreased (Figure 4a). Two of the lower peptide density conditions were chosen and labeled with antibodies, followed by flow cytometry analysis (Figure 4b). Even against a significantly decreased surface peptide density on the magnetic beads (16.7% and 3.33%), the antibodies were not able to distinguish between unmodified, mono-, di-, and trimethylated lysine 9 (Figure 4c). Other buffers were also tested but unable to rescue antibody performance (Supplemental Figure S4). These results suggest that immobilization density alone cannot fully explain the degraded performance of peptide-labeled beads.

### 2.5. Soluble Peptide Binding Followed by Immobilization Improves Specificity and Yield

**Figure 5:**
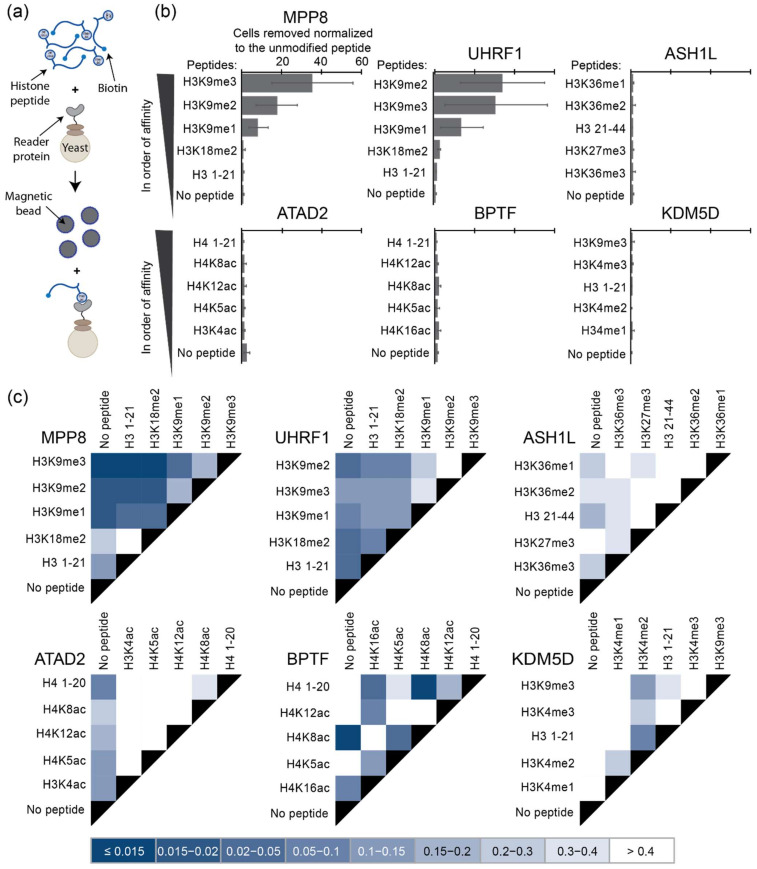
Soluble peptide binding followed by immobilization improves specificity. (**a**) Modified “soluble MACS” method. Interaction between the freely soluble biotinylated peptide and the yeast-displaying protein is then followed by interaction between the peptide–yeast complex and streptavidin-coated magnetic beads. (**b**) Relative amounts of yeasts magnetically separated by modified histones compared to an unmodified peptide control; error bars represent the standard deviation from triplicate samples; peptides displayed in order of binding affinity calculated in Figure 1 and from the literature (see Table 1). (**c**) Discrimination between the amounts of yeast separated via magnetization; darker colors are associated with a higher level of discrimination; the legend indicates p-value comparing each peptide; pairwise comparison via single-factor ANOVA.

**Figure 6:**
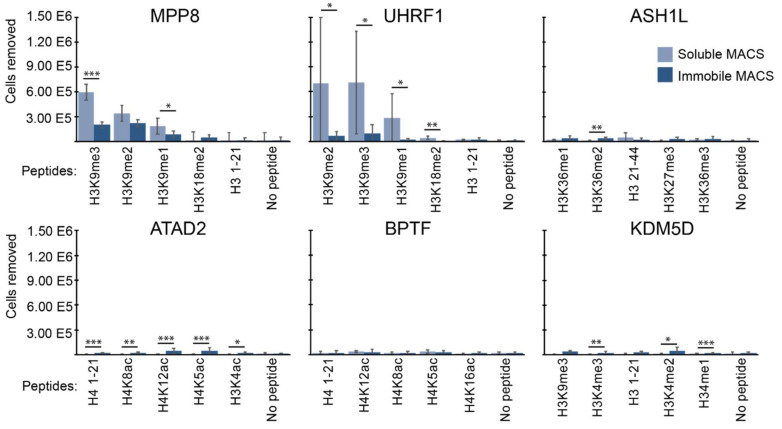
Soluble peptide binding followed by immobilization improves yield. The number of protein-displaying cells pulled down in classic versus soluble MACS methods as a function of histone peptide; error bars represent the standard deviation of triplicate pulldowns; * *p* < 0.1, ** *p* < 0.05, *** *p* < 0.01; *p*-values calculated via single-factor ANOVA.

As another approach to try and mitigate the negative effects of peptide immobilization on magnetic beads, we tested one more method. This method started with soluble peptide labeling of yeast-displaying protein binders (Figure 5a). Once the interaction between freely soluble histone peptides and binding proteins displayed on the yeast surface reached equilibrium, excess unbound peptide was washed away. Then, streptavidin-coated magnetic beads were introduced to the system. This change in the order of protocol steps (“soluble MACS”) reduces potential unwanted avidity effects in the interaction between the displayed protein and the biotinylated peptide [49]. For the binding domains that have higher affinity (MPP8 and UHRF1), soluble MACS was able to moderately increase discrimination between the modified histone peptides (Figure 5b,c). This effect appeared due to a higher yield of cells pulled down in soluble MACS compared to conventional MACS (Figure 6). For those domains with relatively weak affinity towards modified histone peptides (ASH1L, ATAD2, BPTF, and KDM5D), even soluble MACS only slightly improved specificity, suggesting a limitation towards detecting weak binders persists. 

## 3. Discussion

In aggregate, these data suggest that histone peptides do not follow conventional rules when used in traditional protein engineering platforms. This is further exacerbated by the fact that affinity reagents for histone PTMs must discriminate between differentially modified forms of the same amino acid (H3K9, H3K9me1, H3K9me2, H3K9me3, H3K9ac) where the difference can often be just a few atoms. They would preferably also be able to distinguish between the presence or absence of adjacent modifications and very similar amino acid sequence motifs like H3K9 and H3K27 that both are within the A–R–K–S peptide sequence [1,50]. In the face of such requirements, histone tails present several distinct features that only augment the challenge of engineering binding partners in comparison to natural evolution that has had considerably more opportunity to hone such interactions. Histone tails have high charge density, low hydrophobicity, and are intrinsically disordered [1]. The interactions between modified histone tails and natural binding proteins rely more heavily on electrostatic contributions and hydrogen bonding rather than on the complementary and structured hydrophobic surfaces that typically drive protein–protein interactions [1]. This is likely to introduce complications in classic high-throughput techniques for protein engineering. In particular, abundant opportunities for electrostatic and hydrogen bonding interactions could drive nonspecific intermolecular interactions between peptides when brought in close proximity and high density on the surfaces of beads, for example. The mode of attachment of histone peptides to surfaces and their surface density seems to also be critical. It has been shown previously that structural and activity changes are observed upon peptide adsorption to a surface [51]. The hydrophilic nature of modified histone peptides may lead to “hydrophilic aggregation” upon introduction to the hydrophobic surface of polystyrene-based magnetic beads, leading to epitope masking that is not observed while the peptide is in the soluble form [52,53]. Specific classes of histone interactions may also require special considerations and protein engineering systems. For example, the principles underlying interactions with acetylated histones and methylated histones are substantially different, with the former relying on the hydrophobic effect and the latter relying on cation–π interactions and size exclusion by aromatic cages [1,54,55,56,57]. Histone interactions are also often associated with weaker binding affinities [58]. In fact, the rich regulatory landscape of chromatin modifications and interactions is in large part driven by many weak interactions. These weak interactions help stabilize multisubunit protein complexes and, in many cases, confer combinatorial complexity and logic. Weak interactions also enable a more efficient mechanism for proteins to search the genome for their target sites through fast transient interactions [59,60,61]. However, weaker binding affinities are harder to “pan” for protein engineering platforms and can be difficult to identify and characterize using conventional biochemical approaches such as bead-based pulldowns as well [62].

In this work, soluble MACS conditions, which allow for one-to-one interactions between modified histone peptides and proteins displayed on the yeast surface, were more effective in providing this specificity over conventional immobile MACS conditions. However, neither MACS condition could reach the level of specificity achieved by labeling yeast-displayed proteins with soluble histone peptides and analyzing by flow cytometry. Importantly, a broad range of buffer conditions often tuned in other biochemical assays was not able to improve specificity. Future approaches might assess new substrate materials for immobilization of histone peptides with specially tuned chemical properties such as well-defined spacing at the nanoscale between locations of bound peptides to avoid intermolecular interactions or hydrophilic aggregation. While flow cytometric approaches can be used in high-throughput approaches and directed evolution, future advances that enable the use of MACS with histone targets would unlock the throughput and greater coverage of molecular diversity that MACS affords over fluorescence-activated cell sorting. These findings may also have implications for other techniques using immobilized modified histone peptides such as peptide arrays and peptide pulldowns using avidin–agarose beads. The mode of immobilization, immobilization surface properties, and peptide density should be considered as they may have more of an effect on biomolecular recognition specificity than previously considered. These key bottlenecks may explain the dearth of high-throughput approaches applied to the characterization or engineering of histone-binding proteins and affinity reagents. Future work using biophysical, structural biology, and biochemical characterizations could further elucidate the mechanism(s) for the degraded performance of histone peptides on surfaces and could unlock the full use of high-throughput platforms, directed evolution, and combinatorial screening in epigenome engineering. 

## 4. Materials and Methods

### 4.1. Plasmids and Yeast Culture

*Saccharomyces cerevisiae* strain EBY100 was used for all yeast experiments; pCTCON vector contains a TRP selectable marker and pCT302 vector contains a LEU selectable marker. Plasmid DNA was transformed into chemically competent EBY100 using Frozen-EZ yeast transformation Kit II (Zymo Research; Irvine, CA, USA). Trp-deficient SDCAA and SGCAA medium was used for culturing cells and inducing cell surface protein expression for the cells containing the pCTCON vector and Leu-deficient SDSCAA1 (-Leu) and the SGSCAA1 (-Leu) medium was used for cells containing the pCT302-based vector. Leu- and Trp-deficient SDSCAA2 (-Leu/-Trp) and SGSCAA2 (-Leu/-Trp) media were used for cells containing both the pCT302 and pCTCON-based vectors; (-Leu) and (-Leu/-Trp) media have similar composition to SDCAA and SGCAA media except they contain a synthetic dropout mix (1.62 g/L) lacking leucin, or leucine and tryptophan, respectively, instead of casamino acids. Yeast cells were cultured in the SDCAA, SDSCAA1, or SDSCAA2 medium, as appropriate, for 20–24 h at 30 °C with shaking at 250 rpm. Protein expression was induced by transferring cells into the SGCAA, SGSCAA1, or SGSCAA2 medium at an OD_600_ of 1 and cultured for 16–20 h at 20 °C with shaking at 250 rpm. Untransformed EBY100 was grown in the YPD medium for 20–24 h at 30 °C with shaking at 250 rpm.

### 4.2. Plasmid Construction 

All the displayed proteins were encoded as fusions to Aga2p, a yeast cell mating protein. The chromodomain of MPP8, the tandem Tudor domains of UHRF1, the bromo-adjacent homology domain of ASH1L, the bromodomain of ATAD2, the bromodomain of BPTF, and the jmjN domain of KDM5D were all inserted between the NheI and BamHI sites of pCTCON using amplification primers with restriction enzyme-cut sites to generate pCTCON-MPP8, pCTCON-UHRF1, pCTCON-ASH1L, pCTCON-ATAD2, pCTCON-BPTF, and pCTCON-KDM5D. All open reading frames were amplified from cDNA made from a combination of HEK293T, Jurkat, and K562 cells. The amplified proteins and the pCTCON backbones were digested with BamHI and NheI restriction enzymes (New England Biolabs; Ipswich, MA, USA) according to the manufacturer’s protocol. Restriction digests were performed in 20 µL for 1 h at 37 °C. The digested plasmid backbones were treated with rSAP (New England Biolabs; Ipswich, MA, USA) for the final 5 min of the digestion. Ligations of the digested plasmid backbones and PCR products occurred for 5–10 min at RT using T4 DNA ligase (Promega; Madison, WI, USA) prior to transformation into NEB^®^ Turbo Competent *E. coli*; 24-h *E. coli* cultures were harvested for their plasmids using a ZR Plasmid Miniprep-Classic kit; the pCT302–NanoLuc plasmid construction was described previously [35]; the pYD1-mSA (Addgene plasmid #39865) plasmid construction was described previously [46].

### 4.3. Flow Cytometry and Affinity Determination

The equilibrium dissociation constant (K_D_) was determined using cell surface titration as described [63]. Briefly, yeast cells displaying one of the histone-associating proteins were incubated with various concentration of the biotinylated modified histone peptides in 0.1% PBSA (PBS, pH 7.4, 0.1% BSA) at room temperature, followed by streptavidin–PE. Flow cytometric analysis was used to measure the PE fluorescence intensity for each peptide concentration. The binding affinity between each protein–peptide pairing and confidence intervals were estimated as previously described [16].

### 4.4. Magnetic Activated Cell Sorting and Luciferase Quantification

Magnetic beads were functionalized with biotinylated modified histone peptides by incubating 2 µg of biotinylated peptide per 25 µL Dynabeads Biotin Binder Beads (4 × 10^8^ beads/mL, Thermofisher Scientific; Waltham, MA, USA) for 2 h with rotation at RT in 0.1% PBSA. Next, the magnetic beads were washed two times with 0.1% PBSA and blocked in 1% PBSA (PBS, pH 7.4, 1% BSA) for one hour with rotation at RT. Beads not functionalized with a peptide were similarly washed and blocked; 5 × 10^6^ beads were then incubated with 5 × 10^6^ protein- and NanoLuc-displaying yeasts and 5 × 10^8^ EBY100 cells in 2 mL 1% PBSAT (PBS, pH 7.4, 1% BSA, 0.05% Tween-20) for 2 h with rotation at RT. After that, the incubations were placed onto a magnet to isolate any cells bound to the magnetic beads. Other incubations buffers tested along with 1% PBSAT were 1% PBSA and heparin buffer (20 mM HEPES, pH 7.4, 150 mM KCl, 2 mM MgCl_2_, 2 µg/mL heparin). A one-to-one ratio of beads to displaying yeast is described above, and five-to-one and ten-to-one ratios were also tested. EBY100 was always present in 100-fold excess of displaying yeast in all the experimental variations. 

After any cells not bound to the magnetic beads were removed with a magnet, the beads and bound protein-displaying cells were washed gently three times with 1% PBSAT or respective buffer and then resuspended in 100 µL PBS; 100 µL of the Nano-Glo Luciferase Assay system (Promega; Madison, WI, USA) was added to the magnetic bead solution. The reaction was allowed to proceed for three minutes, and then the tube containing the magnetic beads was placed onto a magnet; 100 µL of the reaction was plated in duplicate onto a 96-black-well plate with a clear, flat bottom. The luminescence was read using a Tecan Infinite 200 plate reader using an integration time of 400 ms, settle time of 0 ms, and no attenuation. The standard curves generated using known quantities of protein-displaying cells were used to estimate the number of cells removed with the magnet; p-values were calculated using single-factor ANOVA in Excel. 

### 4.5. Antibody Specificity through Flow Cytometry on Magnetic Beads

Magnetic beads were functionalized with biotinylated modified histone peptides as described above; 5 × 10^6^ beads were then incubated with either an H3K9me1, H3K9me2, or H3K9me3 antibody for 30 min with rotation at 4 °C in 100 µL of the incubation buffer (ab1220 1:200, ab9045 1:200, ab8898 1:250, Abcam; Cambridge, UK). The incubation buffers tested were 0.1% PBSA, 1% PBSA, 1% PBSAT, 50 mM Tris HCl + 10 mM NaCl, 50 mM Tris HCl + 300 mM NaCl, and 50 mM Tris HCl + 500 mM NaCl. The samples were then washed with the incubation buffer and incubated with a secondary antibody for 10 min with rotation at 4 °C in the dark in 100 µL 0.1% PBSA (ab150075 1:250, ab150107 1:250, Abcam; Cambridge, UK). The samples were washed in 0.1% PBSA and run on a MACSQuant VYB cytometer using a 561 nm laser and a 661/20 nm filter. Flow cytometry data were analyzed with the FlowJo software. 

### 4.6. Antibody Specificity through Flow Cytometry on mSA-Displaying Yeast

Prior to labeling, 2 × 10^6^ pYD1-mSA-containing yeast cells were washed and pelleted. The samples were then incubated with either an H3K9me1, H3K9me2, or H3K9me3 antibody for 30 min with rotation at 4 °C in 100 µL of 0.1% PBSA (ab1220 1:200, ab9045 1:200, ab8898 1:250, Abcam; Cambridge, UK). The samples were then washed with 0.1% PBSA and incubated with a secondary antibody for 10 min with rotation at 4 °C in the dark in 100 µL 0.1% PBSA (ab150075 1:250, ab150107 1:250, Abcam; Cambridge, UK). The samples were washed in 0.1% PBSA and run on a MACSQuant VYB cytometer using a 561 nm laser and a 661/20 nm filter. Flow cytometry data were analyzed with the FlowJo software.

### 4.7. Decreasing Peptide Density on Magnetic Beads

Magnetic beads were functionalized with mixtures of biotinylated modified histone peptides and biotin. The total mass of biotin and biotinylated modified histone peptide was kept constant at 1.5 µg. Thirty different ratios were tested. For each ratio, 5 × 10^6^ beads were then incubated with an H3K9me2 antibody for 30 min with rotation at 4 °C in 100 µL of 1% PBSA (ab1220 1:200, Abcam; Cambridge, UK). The samples were then washed with 1% PBSA and incubated with a secondary antibody for 10 min with rotation at 4 °C in the dark in 100 µL 0.1% PBSA (ab150107 1:250, Abcam; Cambridge, UK). The samples were washed in 0.1% PBSA and run on a MACSQuant VYB cytometer using a 561 nm laser and a 661/20 nm filter. Flow cytometry data were analyzed with the FlowJo software. 

Antibody specificity flow as described above was repeated for samples containing 16.7% peptide in both 1% PBSA and 50 mM Tris HCl + 300 mM NaCl and 3.33% peptide in 1% PBSA. 

### 4.8. Soluble Magnetic Activated Cell Sorting and Luciferase Quantification

Biotinylated modified histone peptide (1 µg) was incubated with 5 × 10^6^ protein- and NanoLuc-displaying yeasts and 5 × 10^8^ EBY100 cells in 2 mL 1% PBSAT (PBS, pH 7.4, 1% BSA, 0.05% Tween-20) for 2 h with rotation at RT. The tubes were pelleted and washed to remove any unbound peptides and resuspended in 2 mL 1% PBSAT; 5 × 10^6^ washed magnetic beads were added and incubated with the cells for 10 min with rotation at RT. After that, the incubations were placed onto a magnet to isolate any cells bound to the magnetic beads. Luminescence determination proceeded as previously described. 

## Figures and Tables

**Table 1:** Human protein domains used in experiments along with function and histone PTM binding preferences.

Protein	Domain	Amino Acids	Function	Histone PTM Binding
MPP8 [25,26,27,28]	Chromodomain	49–120	Interacts with H3K9 methyltransferases GLP and ESET and DNA methyltransferase 3A	H3K9 methylation
UHRF1 [26,29,30]	Tandem Tudor domains	127–285	E3 ubiquitin ligase, recruits DNMT1	H3K9 methylation
ASH1L [2,31,32]	Bromo-adjacent domain	2261–2798	H3K36 methyltransferase	H3K36 lower-order methylation
ATAD2 [32,33,34]	Bromodomain (IV)	1001–1071	Interacts with the androgen receptor, estrogen receptor alpha, and E2F transcription factors	H4 acetylation
BPTF [32,34]	Bromodomain (I)	2944–3014	Subunit of the NURF chromatin-remodeling complex	H4 acetylation
KDM5D [2,31]	jmjN domain	14–55	H3K demethylase	H3K higher-order methylation

## Data Availability

The data presented in this study are available on request from the corresponding author.

## Supplementary Materials

The following supporting information can be downloaded at https://www.mdpi.com/article/10.3390/ijms23031691/s1.
