# Supplementary material for: Modified Histone Peptides Linked to Magnetic Beads Reduce Binding Specificity
Source: Int J Mol Sci. 2022 Feb 1;23(3):1691. doi: 10.3390/ijms23031691 (PMC8836101; doi:10.3390/ijms23031691)
Supplement: Supplementary file 1 [file ijms-23-01691-s001.zip › ijms-1553314-supplementary.pdf]

Supplementary Materials

Identifying challenges and opportunities for histone peptides in protein engineering platforms

Jenna N Meanor[1], Albert J Keung[1], Balaji M Rao[1,2]

[1]Department of Chemical and Biomolecular Engineering, North Carolina State University, Campus Box 7905, Raleigh, NC 27606, USA

[2]Golden LEAF Biomanufacturing Training and Education Center (BTEC), North Carolina State University, Raleigh, NC 27695, USA

Correspondence: ajkeung@ncsu.edu, bmrao@ncsu.edu

Contents:

Supplemental Table S1: DNA sequences of protein domains

Supplemental Table S2: Histone peptides

Supplemental Table S3: Fluorophore conjugated secondary antibodies and streptavidin

Supplemental Table S4: Primary antibodies

Supplemental Figure S1 MACS with yeast displaying NanoLuc

Supplemental Figure S2 MACS with yeast displaying MPP8 under varying buffer conditions and yeast-to-bead ratios

Supplemental Figure S3 Antibody labeling of histone peptides presented on magnetic beads

Supplemental Figure S4 Antibody labeling of low density histone peptides presented on magnetic beads

Supplemental Table S1 DNA sequences of protein domains

Protein	Domain	DNA Sequence
MPP8	Chromodomain	GGCGACAGTGAGGAGGACGGAGAGGATGTGTTCGAGGTGGAGAAGATCC<br>TGGACATGAAGACCGAGGGGGGTAAAGTTCTTTACAAAGTTCGCTGGAAA<br>GGCTATACATCGGATGATGATACCTGGGAGCCCGAGATTCACCTGGAGGA<br>CTGTAAAGAAGTGCTTCTTGAATTTAGGAAGAAAATTGCAGAGAACAAAGC<br>CAAAGCAGTCAGGAAG
UHRF1	Tandem Tudor domains	GACATGTGGGATGAGACGGAATTGGGGCTGTACAAGGTCAATGAGTACGT<br>CGATGCTCGGGACACGAACATGGGGGCGTGGTTTGAGGCGCAGGTGGTC<br>AGGGTGACGCGGAAGGCCCCCTCCCGGGACGAGCCCTGCAGCTCCACGT<br>CCAGGCCGGCGCTGGAGGAGGACGTCATTTACCACGTGAAATACGACGA<br>CTACCCGGAGAACGGCGTGGTCCAGATGAACTCCAGGGACGTCCGAGCG<br>CGCGCCCGCACCATCATCAAGTGGCAGGACCTGGAGGTGGGCCAGGTGG<br>TCATGCTCAACTACAACCCCGACAACCCCAAGGAGCGGGGCTTCTGGTAC<br>GACGCGGAGATCTCCAGGAAGCGCGAGACCAGGACGGCGCGGGAACTCT<br>ACGCCAACGTGGTGCTGGGGGATGATTCTCTGAACGACTGTCGGATCATC<br>TTCGTGGACGAAGTCTTCAAGATTGAGCGG
ASH1L	BAH	TTGCTGCTTCGTCAGGGTGACTGTGTGTATCTGATGAGGGATAGTCGGCG<br>CACCCCTGATGGCCACCCGGTCCGTCAGTCCTATCGACTGTTATCTCACAT<br>TAACCGAGATAAACTTGACATCTTTCGCATTGAGAAGCTTTGGAAGAATGAA<br>AAAGAGGAACGGTTTGCCTTTGGTCACCATTATTTCCGTCCCCACGAAACA<br>CACCACTCTCCATCCCGTCGGTTCTATCATAATGAACTATTTCGGGTGCCA<br>CTCTATGAGATCATTCCCTTGGAGGCTGTAGTGGGGACCTGCTGTGTGTTG<br>GACCTTTATACGTATTGTAAAGGGAGACCCAAAGGAGTAAAGGAGCAAGAT<br>GTGTACATCTGTGATTATCGGCTTGACAAGTCAGCACACCTGTTTTACAAGA<br>TCCAC
ATAD2	Bromodomain (IV)	GCTATTGACAAGCGATTCCGAGTGTTTACTAAGCCTGTTGACCCTGATGAG<br>GTTCCTGATTATGTCACTGTAATAAAGCAACCAATGGACCTTTCATCTGTAA<br>TCAGTAAAATTGATCTACACAAGTATCTGACTGTGAAAGACTATTTGAGAGA<br>TATTGATCTAATCTGTAGTAATGCCTTAGAATACAATCCAGATAGAGATCCT<br>GGAGAT
BPTF	Bromodomain (I)	CAGGCCCATAAGATGGCCTGGCCTTTCCTTGAACCAGTAGACCCTAATGAT<br>GCACCAGATTATTATGGTGTTATTAAGGAACCTATGGACCTTGCCACCATG<br>GAAGAAAGAGTACAAAGACGATATTATGAAAAGCTGACGGAATTTGTGGCA<br>GATATGACCAAAATTTTTGATAACTGTCGTTACTACAATCCAAGTGACTCCC<br>CATTTTAC
KDM5D	HDM-JARID	TGCCCGGTTTTTGAGCCTAGCTGGGCTGAATTCCAAGACCCGCTTGGCTAC<br>ATTGCGAAAATAAGGCCCATAGCAGAGAAGTCTGGCATCTGCAAAATCCGC<br>CCACCCGCGGATTGGCAGCCTCCT

Supplemental Table S2 Histone peptides

Peptide	Sequence	Manufacturer	Catalog #
H3 1-21	ARTKQTARKSTGGKAPRKQLA - GG - K(BIOTIN) - NH2	Anaspec	AS-61702
H3 21-44	ATKAARKSAPATGGVKKPHRYRPG-GK(Biotin)	Anaspec	AS - 64440-025
H3K4Ac	ART-Kac-QTARKSTGGKAPRKQLA - GGYK(Biotin) - NH2 (1-21)	Active motif	81039
H3K4me1	ART-K(me1)-QTARKSTGGKAPRKQLA-GGK(Biotin)	Anaspec	AS - 64355 - 025
H3K4me2	ART - K(me2) - QTARKSTGGKAPRKQLA - GGK(Biotin)	Anaspec	AS-64356-025
H3K4me3	ART-K(me3)-QTARKSTGGKAPRKQLA-GGK(Biotin)	Anaspec	AS - 64357 - 025
H3K9me1	ARTKQTAR - K(me1) - STGGKAPRKQLA - GGK(Biotin)	Anaspec	AS-64358-025
H3K9me2	ARTKQTAR - K(me2) - STGGKAPRKQLA - GGK(Biotin)	Anaspec	AS-64359-1
H3K9me3	ARTKQTAR - K(me3) - STGGKAPRKQLA - GGK(Biotin)	Anaspec	AS-64360-025
H3K18me2	ARTKQTARKSTGGKAPR - K(me2) - QLA - GGK(Biotin) - NH2	Anaspec	AS-64620-025
H3K27me3	ATKAAR-K(Me3)-SAPATGGVKKPHRYRPG-GK(Biotin)	Anaspec	AS - 64367-025
H3K36me1	ATKAARKSAPATGGV - K(me1) - KPHRYRPG - GK(Biotin)	Anaspec	AS - 64368 - 025
H3K36me2	RKSAPATGGV - K(me2) - KPHRYRPGTV - K(BIOTIN)	Anaspec	AS-64601-025
H3K36me3	ATKAARKSAPATGGV-K(me3)-KPHRYRPG-GK(Biotin)	Anaspec	AS - 64441 - 025
H4 1-20	SGRGKGGKGLGKGGAKRHRKVLRGG-YK(Biotin)-NH2	Active motif	81109
H4K5ac	SGRG - K(Ac) - GGKGLGKGGAKRHRKVLRDNGSGS - K(Biotin)	Anaspec	AS-65229-1
H4K8ac	SGRGKGG - K(Ac) - GLGKGGAKRHRKVLRDNGSGS - K(Biotin)	Anaspec	AS-65230-1
H4K12ac	SGRGKGGKGLG - K(Ac) - GGAKRHRKVLRDNGSGS - K(Biotin)	Anaspec	AS-65208-1
H4K16ac	SGRGKGGKGLGKGGA - K(Ac) - RHRKVLRDNGSGS - K(Biotin)	Anaspec	AS-65209-1

Supplemental Table S3 Fluorophore conjugated secondary antibodies and streptavidin

Secondary Antibody	Manufacturer	Catalog #	Dilution Used
Goat anti-Chicken IgY (H&L) DyLight®488 Conjugate	Immunoreagents	GtxCk-003-D488NHSX	1:250
Donkey anti-Rabbit IgG (H&L) DyLight®633 Conjugate	Immunoreagents	DkxRb-003-E633NHSX	1:250
Donkey anti-Rabbit IgG (H&L) AlexaFluor®647 Conjugate	Abcam	ab150075	1:250
Donkey anti-Mouse IgG (H&L) AlexaFluor®647 Conjugate	Abcam	ab150107	1:250
Streptavidin, R-Phycoerythrin Conjugate (SAPE)	ThermoFisher	S866	1:250

Supplemental Table S4 Primary antibodies

Antibody Target	Manufacturer	Catalog #	Host Species	Dilution Used
HA	Thermo Fischer Scientific	PA1-985	Rabbit	1:100
C-Myc	Thermo Fischer Scientific	A-21181	Chicken	1:100
H3K9me1	Abcam	ab9045	Rabbit	1:200
H3K9me2	Abcam	ab1220	Mouse	1:200
H3K9me3	Abcam	ab8898	Rabbit	1:200

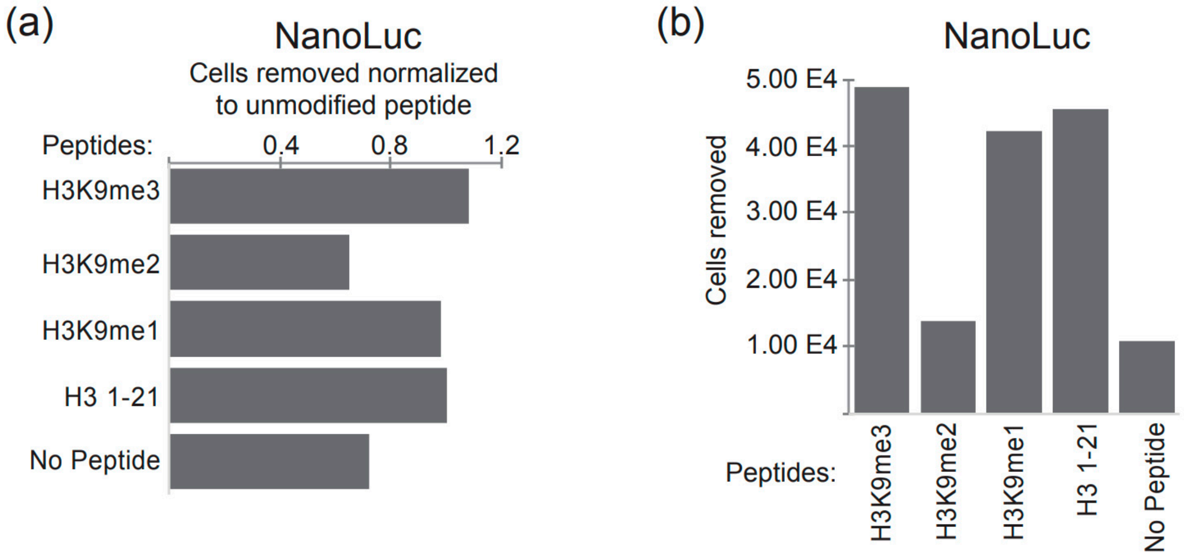

Supplemental Figure S1. MACS with yeast displaying NanoLuc (**a**) Relative amount of yeast displaying NanoLuc magnetically separated by beads linked to modified histone peptides compared to an unmodified histone peptide control; (**b**) The number of yeast displaying NanoLuc pulled-down in classic MACS as a function of histone peptide

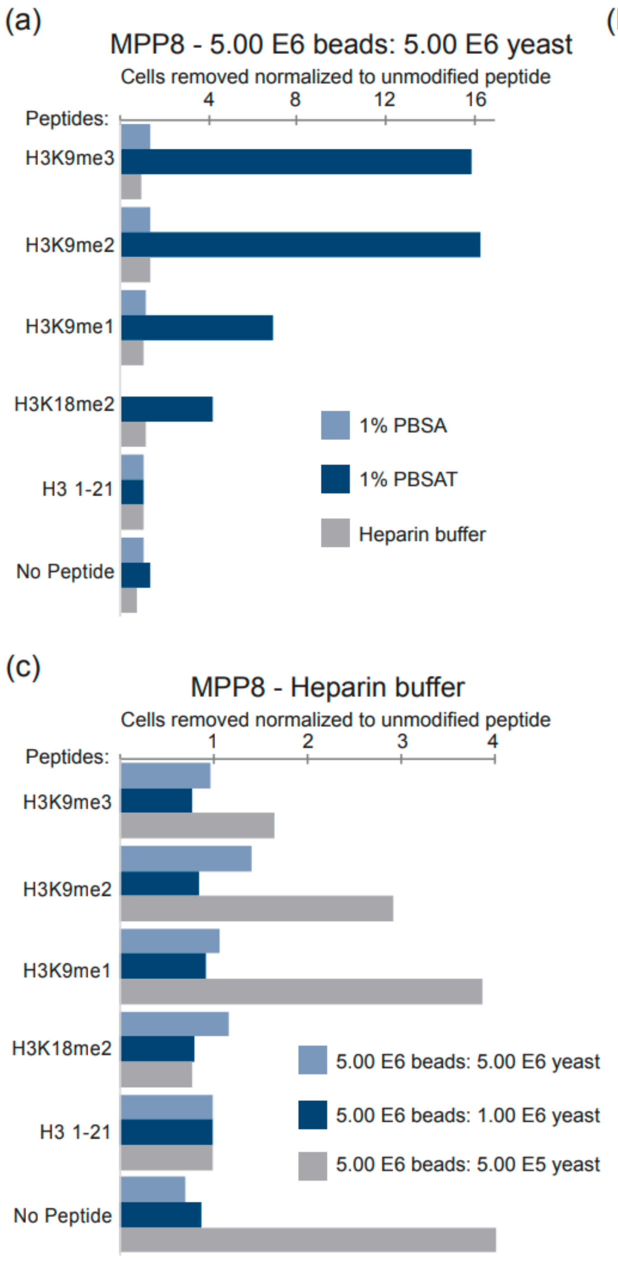

Supplemental Figure S2 MACS with yeast displaying MPP8 under varying buffer conditions and yeast-to-bead ratios (**a**) Relative amount yeast displaying MPP8 magnetically separated by histone peptide linked to magnetic bead compared to an unmodified peptide control for 1% PBSA, 1% PBSAT, and heparin buffer at a yeast to beads ratio of one-to-one; (**b**) Relative amount of MPP8 displaying yeast magnetically separated by histone peptide linked to magnetic bead compared to an unmodified peptide control in 1% PBSAT for yeast to beads ratios of one-to-one and one-to-five **c** Relative amount of MPP8 displaying yeast magnetically separated by histone peptide linked to magnetic bead compared to an unmodified peptide control in heparin buffer for yeast to beads ratios of one-to-one and one-to-five, and one-to-ten.

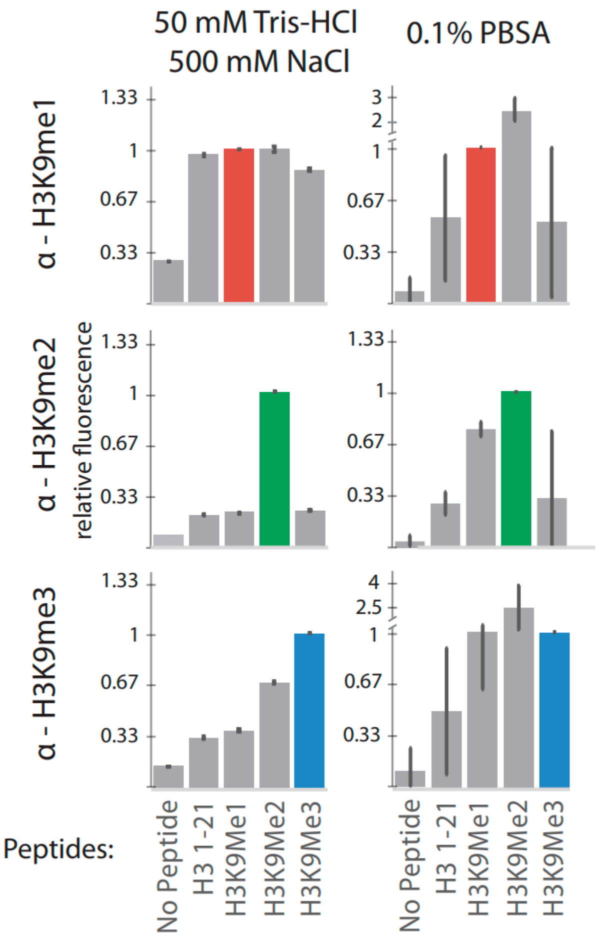

Supplemental Figure S3 Antibody labeling of histone peptides presented on magnetic beads. Relative fluorescence of each peptide–antibody pairing. The pairings with expected specific interactions with each antibody are indicated by colored bars; error bars represent standard deviation from triplicate samples.

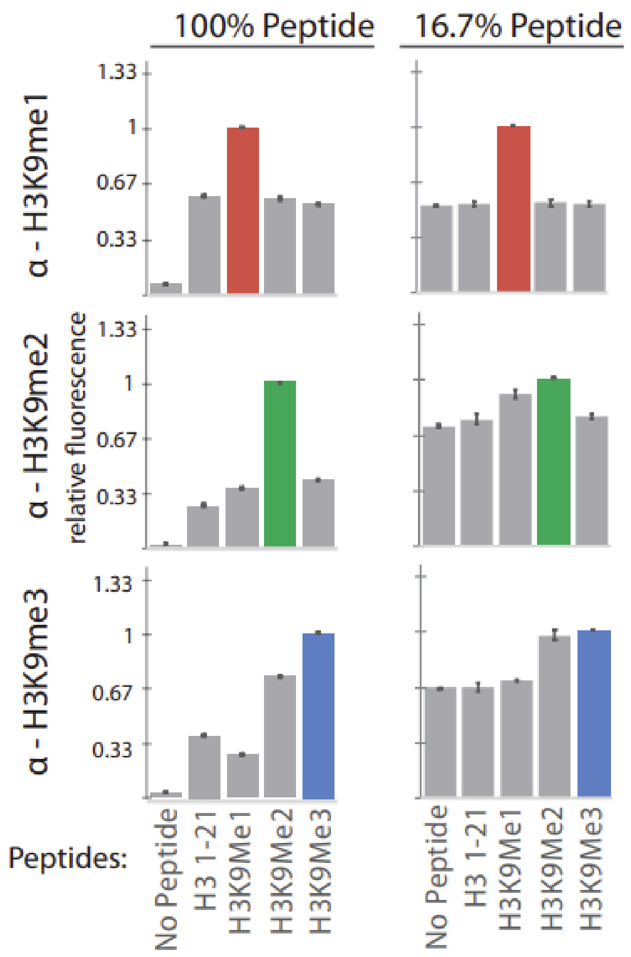

Supplemental Figure S4 Antibody labeling of low density histone peptides presented on magnetic beads. Relative fluorescence of each peptide–antibody pairing. The pairings with expected specific interactions with each antibody are indicated by colored bars. Buffer used was 50 mM Tris HCl 300 mM NaCl; error bars represent standard deviation from triplicate samples.
